# Role of FoxO transcription factors in aging and age-related metabolic and neurodegenerative diseases

**DOI:** 10.1186/s13578-021-00700-7

**Published:** 2021-11-02

**Authors:** Shuqi Du, Hui Zheng

**Affiliations:** Huffington Center on Aging, Baylor College of Medicine, Houston, TX USA

**Keywords:** Aging, FoxO, Age-related disease, Type 2 diabetes mellitus, Alzheimer’s disease

## Abstract

Aging happens to all of us as we live. Thanks to the improved living standard and discovery of life-saving medicines, our life expectancy has increased substantially across the world in the past century. However, the rise in lifespan leads to unprecedented increases in both the number and the percentage of individuals 65 years and older, accompanied by the increased incidences of age-related diseases such as type 2 diabetes mellitus and Alzheimer’s disease. FoxO transcription factors are evolutionarily conserved molecules that play critical roles in diverse biological processes, in particular aging and metabolism. Their dysfunction is often found in the pathogenesis of many age-related diseases. Here, we summarize the signaling pathways and cellular functions of FoxO proteins. We also review the complex role of FoxO in aging and age-related diseases, with focus on type 2 diabetes and Alzheimer’s disease and discuss the possibility of FoxO as a molecular link between aging and disease risks.

## Introduction

The FoxO transcription factor family belongs to the Forkhead box transcription factors consisting of DAF-16 in *Caenorhabditis elegans*, dFOXO in *Drosophila Melanogaster*, and four members in mammals: FoxO1, FoxO3, FoxO4 and FoxO6. FoxO proteins receive and integrate various upstream signals and, through post-translational modifications and cytoplasmic-to-nucleus trafficking, transcriptionally control a wide range of downstream targets in both peripheral tissues and the central nervous system (CNS). Of particular significance, FoxO is an integral component of the insulin and insulin-like growth factor signaling pathway that plays essential and evolutionarily conserved functions in cellular metabolism, aging and longevity. Dysfunction of this pathway is a major cause of type 2 diabetes mellitus (T2DM), a metabolic disorder influenced by age. Interestingly, accumulating evidence indicates that T2DM is associated with an increased risk of developing Alzheimer’s disease (AD), which is the most common cause of dementia in the elderly. Recognized as a neurodegenerative disorder, AD is also accompanied by significant metabolic disturbances in the brain. Given its prominent role in metabolic homeostasis and organismal longevity, misregulation of the FoxO signaling pathway may underlie both age-associated functional decline and age-related diseases. This review highlights the diverse functions of FoxO in physiology, aging and disease. We will start by describing the molecular characteristics of the FoxO proteins, followed by their cellular signaling pathways and associated activities. Next we will review their physiological functions, with particular emphasis on aging and lifespan regulation across species and CNS function in the mammalian system. Lastly we will summarize the published literature highlighting a critical role of FoxO in age-associated metabolic and neurodegenerative diseases, in particular T2DM and AD, and provide our perspective on how these two diseases with distinct clinical presentations may be connected through FoxO signaling and regulation.

## FoxO transcription factors

### FoxO proteins and functional domains

**Fig. 1:**
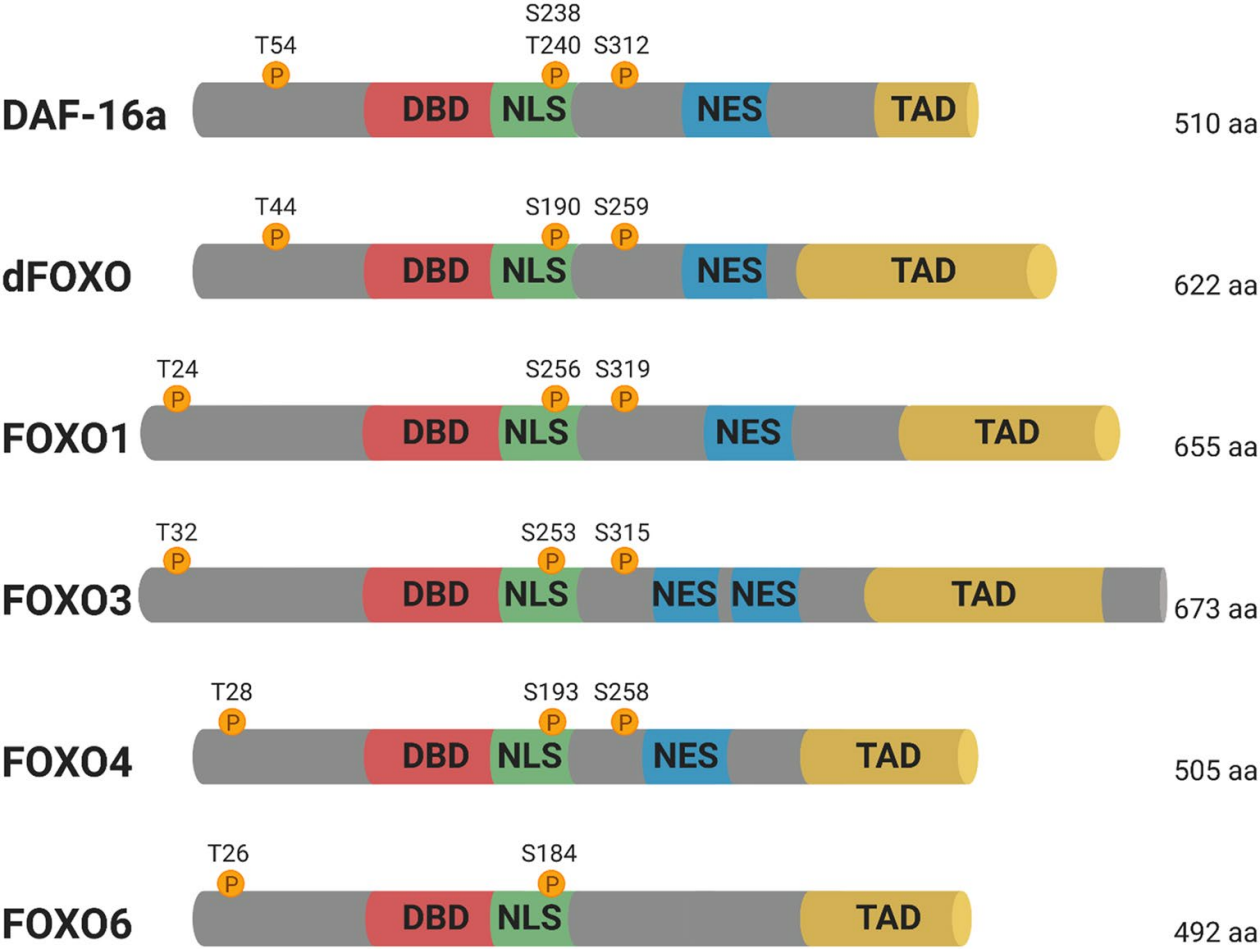
Structural domains of FoxO proteins across species. Members in FoxO family share a highly conserved forkhead DNA-binding domain (DBD), a nuclear localization signal (NLS), one or two nuclear export signal (NES), and a transactivation domain (TAD). FoxO6 does not have a functional NES. The positions of Akt phosphorylation sites are indicated by circles. The first two phosphorylation sites also serve as binding motifs for 14-3-3 protein [2–8])

The FoxO proteins have a conserved winged-helix DNA-binding domain (DBD) known as the Forkhead domain. They bind to DNA duplexes as monomers and recognize two consensus sequences, the DAF-16 family member-binding element (DBE) 5′-GTAAA(T/C)AA-3′ and the insulin responsive element (IRE) 5′-(C/A)(A/C)AAA(C/T)AA-3′ [1]. In addition to the DBD, FoxO proteins also carry a nuclear localization signal, a nuclear export signal, and a C-terminal transactivation domain (Fig. 1). These functional structures together with numerous post-translational modifications (PTMs) tightly control FoxO’s subcellular localization and transcription activity and afford its sophisticated regulation of various biological processes.

## FoxO signaling pathways

**Fig. 2:**
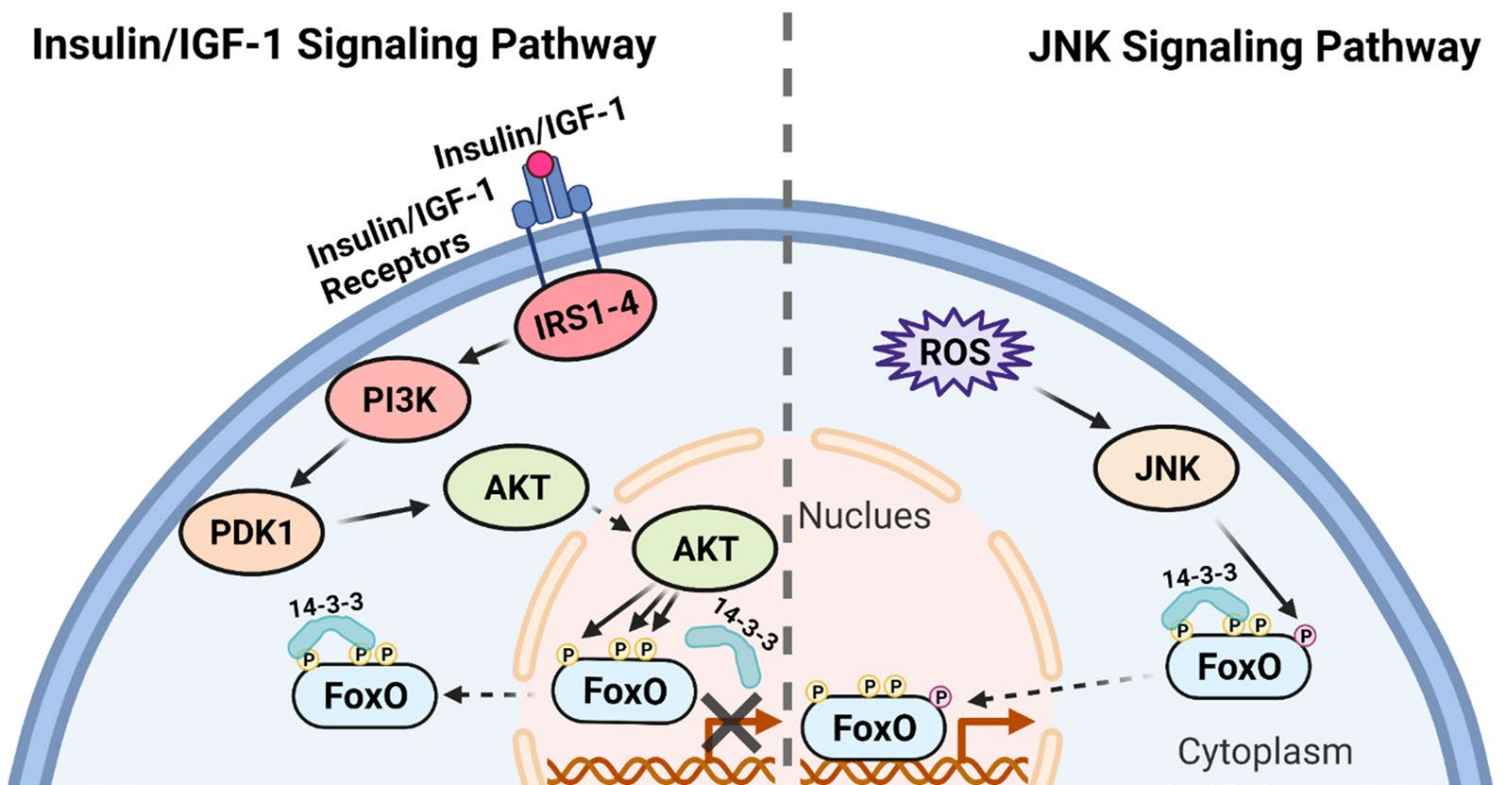
Evolutionarily conserved Insulin/IGF-1 signaling pathway and JNK signaling pathway for FoxO regulation. The binding of Insulin or IGF-1 to the receptors trigger a kinase cascade, which results in FoxO phosphorylation, 14-3-3 binding and nuclear export (left). Cellular ROS stimulates JNK, which phosphorylates and activates FoxO, leading to its nuclear transport (right) [24]

FoxO activity is mainly regulated by its PTMs and subsequent subcellular translocation between the cytosol and the nucleus, where it transcriptionally controls a wide range of downstream targets. In addition, FoxO PTMs also influence its binding affinity with its cofactors, resulting in distinct transcription profiles [9]. Two evolutionarily conserved pathways exist to regulate FoxO activities in cells (Fig. 2). The canonical insulin and growth factor signaling initiates when secreted insulin or insulin-like growth factors (IGFs) bind to their cell surface receptors. Dimerized receptors trigger a series of autophosphorylation and recruit insulin receptor substrate 1–4 (IRS1-4) and phosphatidylinositol 3-kinase (PI3K), the latter increases the local concentrations of phosphatidylinositol (3,4,5)-trisphosphate (PIP3). PIP3 acts as a second messenger to activate phosphoinositide-dependent kinase 1 (PDK1) and protein kinase B (AKT or PKB). Active AKT translocates to the nucleus and phosphorylates FoxO at three conserved residues, enhancing the binding of FoxO proteins to 14-3-3 and leading to their cytoplasmic localization. Inactivation of FoxO favors cellular growth under normal conditions. The other pathway involves FoxO’s role in stress response. When cells are in a stressed condition, such as increasing levels of reactive oxygen species (ROS), c-Jun N-terminal kinase (JNK) is activated and phosphorylates cytoplasmic FoxO. This stimulatory phosphorylation induces the release of FoxO from 14-3-3 and upregulates its transcriptional activity. Importantly, the opposing regulation of FoxO activity by insulin/IGF pathway and JNK pathway is evolutionarily conserved in *C. elegans*, *Drosophila* and vertebrates [10, 11].

In addition to the two well-established mechanisms described above, numerous other signaling events affect FoxO activity. For example, AMP-activated protein kinase (AMPK), an enzyme stimulated by a high AMP to ATP ratio, phosphorylates and activates FoxO for stress resistance [12]. Surprisingly, this phosphorylation event of FoxO does not alter their subcellular localization. Meanwhile, cyclin-dependent kinases (CDKs) activates FoxO to control the expression of many essential cell cycle components during proliferation [13, 14]. Furthermore, regulation of FoxO activities by ataxia telangiectasia mutated (ATM) and ATM Rad3-related (ATR) proteins have been reported in response to DNA damage [15]. However, the regulation of FoxO goes beyond phosphorylation. In fact, other PTMs are also involved to modulate FoxO functions. The effect of acetylation on FoxO proteins is controlled by the histone acetyltransferases and histone deacetylases (HDACs). Studies have shown that HDAC-mediated deacetylation of FoxO leads to its nuclear translocation and transcriptional activation under nutrient deprivation [16]. On the other hand, the degradation of cytoplasmic FoxO proteins rely on the ubiquitin–proteasome pathway. E3 ubiquitin ligase S-phase kinase associated protein 2 (SKP2) and mouse double minute 2 homolog (MDM2) recognizes FoxO with certain inhibitory phosphorylation or acetylation signals and induces their polyubiquitination for degradation [17, 18]. Under stress conditions like accumulative ROS in cells, MDM2 promotes FoxO mono-ubiquitination as well [19]. Instead of promoting degradation, this modification has a stimulatory effect on FoxO. Lastly, methylation by protein arginine methyltransferase 1/6 (PRMT1/6) and O-GlcNacylation by O-GlcNAc transferase (OGT) have been characterized to impact FoxO activity by blocking the addition of other functional PTMs, adding another layer of complexity to FoxO regulation [20–23].

## Cellular functions of FoxO

**Fig. 3:**
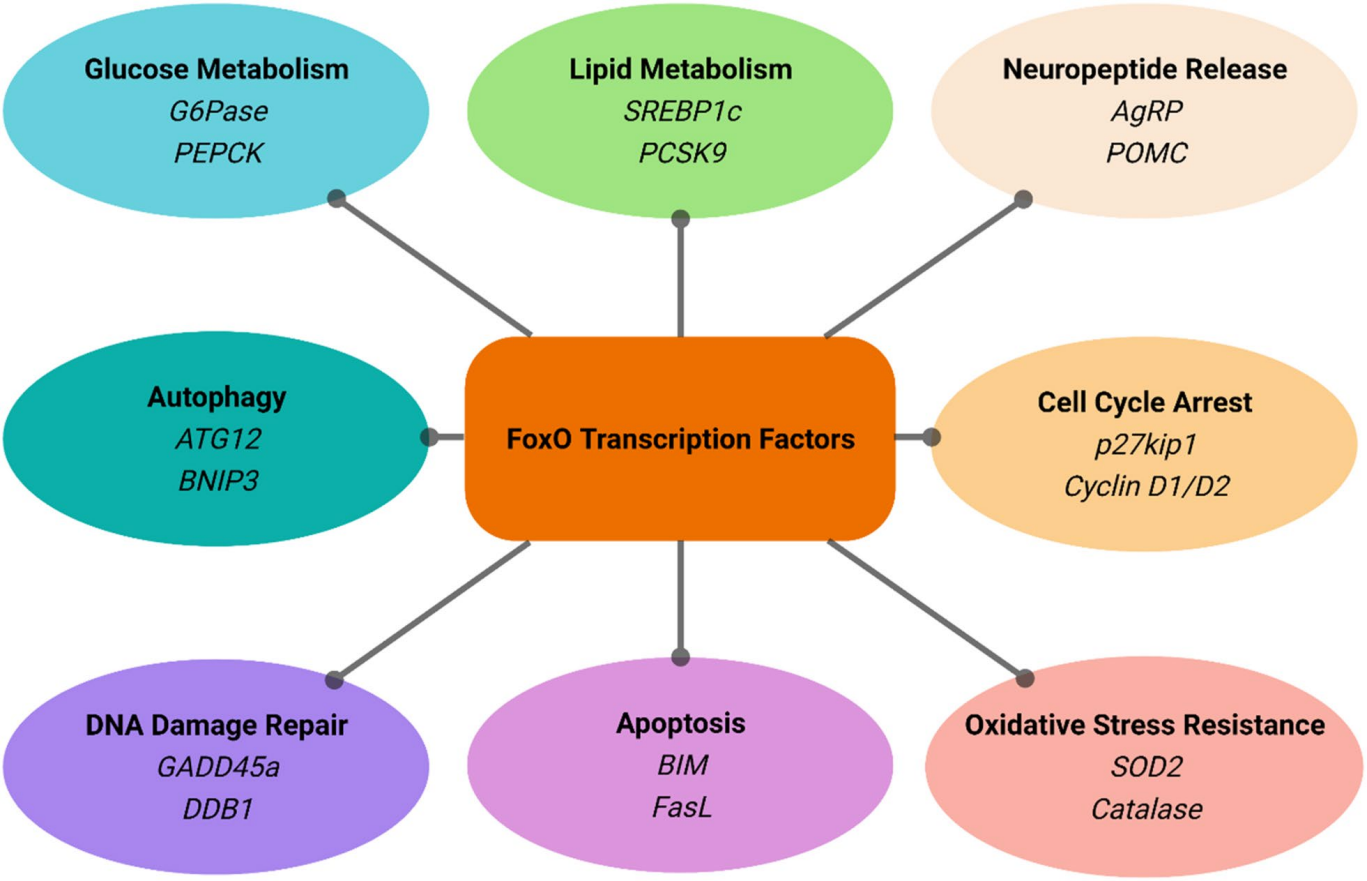
Diverse functions of FoxO. Major FoxO functions are listed here to demonstrate the involvement of FoxO proteins in a variety of cellular processes. These include glucose metabolism, lipid metabolism, oxidative stress resistance, cell cycle arrest, DNA damage repair, energy homeostasis, autophagy, and apoptosis. Select transcriptional targets of FoxO proteins within each pathway are shown

Since their first identification as chromosomal translocation partners in acute myeloid leukemia and alveolar rhabdomyosarcomas, FoxO functions have been extensively studied in cell culture systems [25, 26]. These efforts revealed FoxO as versatile regulators of many essential cellular processes including metabolism, autophagy, cell cycle arrest, DNA damage repair, apoptosis as well as oxidative stress resistance (Fig. 3). Importantly, although FoxO proteins share highly conserved consensus binding sequences, they not only exhibit distinct regulatory features, but also show high cell-type specificity. As most other transcription factors, the binding of FoxO to coactivators or corepressors and the binding of these complexes to other gene regulators ultimately determine the outcome of the gene regulation. In consistency, knockout studies of FoxO members revealed distinct phenotypes. Deletion of *Foxo1* leads to embryonic lethality due to defects in angiogenesis during early development. *Foxo3* knockout mice are viable, but females become infertile after 15 weeks because of the premature primordial follicle activation and subsequent depletion. *Foxo6* deficiency attenuates hepatic gluconeogenesis and impairs memory consolidation. Loss of *Foxo4*, however, does not result in overt anomalies in mice [27, 28]. Therefore, it is crucial to delineate individual FoxO functions in a context-dependent manner [29].

### FoxO regulation of metabolism

Insulin is a peptide hormone secreted by beta cells in the pancreatic islets under high blood glucose conditions. It has a potent effect on promoting the cellular uptake and utilization of glucose and stimulating anabolic pathways like glycogenesis, lipogenesis, and protein synthesis. As a critical downstream effector of the insulin signaling pathway, FoxO proteins respond to the changing nutrients and regulate the metabolic homeostasis in various tissues and organs. Liver is highly sensitive to insulin and its proper physiological function depends on FoxO’s regulation. In hepatocytes, active FoxO1, FoxO3 and FoxO6 induce the expression of two key enzymes in gluconeogenesis, glucose-6-phosphatase (G6Pase) and phosphoenolpyruvates carboxykinase (PEPCK) [30–34]. Consistently, knockout of either *Foxo1* alone or *Foxo1/3/4* altogether specifically in mouse liver leads to lower blood glucose levels under both fasting and non-fasting conditions [35, 36]. *Foxo6* germline knockout mice also exhibit reduced levels of fasting and non-fasting blood glucose [28]. Additionally, FoxO proteins inhibit glycolysis through suppression of glucokinase and pyruvate kinase gene expression [37, 38]. Taken together, liver FoxO contributes to the maintenance of normal blood glucose. Furthermore, FoxO1 is activated in skeletal muscle following strong stress conditions such as starvation, streptozotocin-induced diabetes, or exercise, where it induces the expression of pyruvate dehydrogenase kinase 4 (PDK4) to conserve glucose and gluconeogenesis substrate and decrease the glycolytic flux during energy deprivation [39–41].

Besides glucose metabolism, FoxO also plays an important role in lipid homeostasis by regulating lipogenesis, fatty acid oxidation, lipid transport and cholesterol metabolism. FoxO1 transcriptionally suppresses the lipogenic master regulator sterol regulatory element binding protein 1c (SREBP1c) in the liver and regulates the expression of several genes in fatty acid biosynthesis pathway such as fatty acid synthase, acetyl-CoA carboxylase alpha and stearoyl-CoA desaturase I (*Scd1*) [42–44]. As well, FoxO activates the expression of lipolysis and fatty acid oxidation genes including lipoprotein lipase (LPL) and carnitine palmitoyltransferase-1 (CPT1) [40, 44]. Interestingly, FoxO can also promote breakdown of lipid droplets through lipophagy, an autophagy subtype that selectively targets lipid contents, by inducing the transcription of autophagy-related genes like *ATG5*, *ATG12*, *ATG14* and *BECN1* [45, 46]. In addition, FoxO1 drives the secretion of liver triglycerides into plasma. It is achieved by direct promoter binding and transcriptionally elevating the expression of apolipoprotein ApoC-III [47, 48]. On the other hand, fatty acid uptake and subsequent oxidation are under the control of FoxO1 in muscle cells, where its expression alters the subcellular distribution of fatty acid translocase (FAT/CD36) [49]. In line with the regulation of the fatty acid pathway, FoxO3 suppresses the expression of SREBP-2, the master regulator of cholesterol biosynthesis [50]. It can also promote the low-density lipoprotein (LDL)-cholesterol breakdown by downregulating the level of proprotein convertase subtilisin/kexin type 9 (PCSK9) to preserve the LDL receptor (LDLR)-mediated clearance activity [51]. Meanwhile, FoxO1 regulates cholesterol conversion to bile acids by modulating bile acid biosynthetic genes like *CYP7A1* and *CYP8B1* [52, 53].

The impact of FoxO on metabolic regulation goes beyond peripheral organs. Researchers have observed FoxO1 expression in hypothalamus, the food intake control center in the brain, where it regulates the expression of neuropeptides and affects appetite. It receives and integrates signals of nutritional status and responds by the release of neuropeptides, such as proopiomelanocortin (POMC) and agouti-related peptides (AgRP) [54]. Within the hypothalamus, the arcuate nucleus (ARC) is the “first-order center” for food intake regulation. It contains two groups of neurons, anorexigenic POMC neurons and orexigenic AgRP neurons [55]. FoxO1 is expressed in both types of neurons, and it is located in the nucleus during starvation but translocated to the cytoplasm after feeding [56]. FoxO1 stimulates appetite by direct promoter binding to *Agrp* and *Pomc* genes, with an activation effect on AgRP but inhibiting POMC expression [57, 58]. Consistent with this finding, mice with a hypothalamic-specific knock-in allele overexpressing *Foxo1* showed increased food intake and decreased energy expenditure [59].

### FoxO regulation of autophagy

Autophagy is an evolutionarily conserved self-degradative process through which damaged organelles or macromolecules are broken down and recycled within the cell. Autophagy provides cells with alternative energy sources at critical times during development or under nutrient stress, maintains cellular proteostasis, and eliminates invading pathogens. Three forms of autophagy have been identified based on the way cellular components are sequestered and degraded. Microautophagy is characterized by direct lysosomal engulfment of cytoplasmic molecules. Chaperon-mediated autophagy is a selective degradation pathway which involves HSC70-dependent chaperone recognition of specific cargo proteins and subsequent delivery to the lysosome compartment. Macroautophagy (herein referred to as autophagy) is the most well-studied form and it encapsulates cytoplasmic materials with a special structure called autophagosome that fuses with lysosome for cargo degradation. The autophagy process is divided into different stages based on the presence of some featured structures. Upon activation, autophagy is initiated with the formation of a crescent-shaped double-membrane structure called phagophore. Phagophore grows around and sequesters target components and becomes an autophagosome. The autophagosome then fuses with a lysosome and turns into an autolysosome, where sequestered contents are degraded. The successful completion of these sequential steps involves a number of conserved autophagy-related (ATG) proteins and other autophagy regulators [60].

The autophagy regulatory role of the FoxO family was first described in studies of muscle atrophy in murine models, in which FoxO1 and FoxO3 were found to elevate the autophagic flux by increasing the expression of autophagy genes such as *Ulk2*, *Becn1*, *Lc3b*, *Atg12*, *GabarapL1* and *Bnip3* [61, 62]. Besides its transcriptional activity, Zhao and colleagues demonstrated that cytosolic FoxO1 was responsible for the autophagy induction in human cancer cell lines under oxidative stress or serum starvation. Specifically, SIRT2 acetylates FoxO1 upon stress and the acetylated FoxO1 binds to ATG7 to trigger the autophagy machinery [63]. This discovery was unexpected, as under environmental conditions that favor autophagy induction, FoxO proteins are more likely present in the nucleus. In a follow-up study, they also found that FoxO3 could induce autophagy under the same settings but in a FoxO1-dependent way. It transcriptionally activates the expression of upstream regulator PIK3CA, which then results in FoxO1 phosphorylation and nuclear export [64]. In addition to general autophagy, FoxO3 is involved in the regulation of mitophagy, an autophagy subtype that selectively targets mitochondria. This pathway is mediated by PINK1, a central mitophagy regulator and contributes to the redox homeostasis in the cells [65–67].

### FoxO regulation of other cellular pathways

Other than autophagy and metabolism, FoxO transcription factors are also well known for their regulation in processes like cell cycle arrest, DNA damage repair, apoptosis and oxidative stress resistance. The activation of FoxO, either by transcriptional overexpression or pharmacologically targeting upstream regulators, resulted in a robust cell cycle arrest in cancer cell lines of colon carcinoma, glioblastoma, osteosarcoma, and acute T cell leukemia [68–70]. This FoxO-dependent proliferation regulation is mediated through transcription of multiple cell cycle kinase inhibitors like p27^kip1^ and downregulation of the expression of cyclin D1 and D2 [71–73]. In addition, FoxO3 can stimulate the expression of GADD45a or DDB1 for DNA damage repair during paused division [74, 75]. Besides cell cycle control, FoxO proteins are well-described inducers of apoptosis in different cell types. They can drive the expression of many pro-apoptotic genes including *FASL*, *TRAIL*, *TNFR*, *BIM* and *BMF*, leading to programmed cell death in both normal and cancer cells [76]. The importance of this function is implicated in the fact that a variety of cancers inactivate FoxO to promote their survival. In addition, FoxO proteins have also been intensively studied in their role of oxidative stress response. They act to enhance cellular detoxification via the induction of superoxide dismutase 2 (SOD2) and catalase expression. By protecting cells from ROS accumulation, FoxO reduces the risk of cancer development [77]. Other examples of FoxO functions include the negative regulation of RUNX2 by FoxO1 and FoxO4, which mitigated the migration and invasion of prostate cancer [78, 79]. Studies have also found that FoxO3 deficiency led to the activation of inflammatory NFκB pathway and increased tumor burden in mouse colon [80, 81].

## Physiological functions of FoxO

### FoxO in aging and longevity

#### C. elegans

In 1990s, longevity studies in *C. elegans* led to the identification of the insulin/insulin-like growth factor-1 signaling (IIS) pathway as the first established lifespan-regulating signaling pathway in animals [82]. To be specific, genetic mutations that nullify the activity of DAF-2, the invertebrate insulin/insulin-like growth factor-1 receptor, more than double the lifespan of the worms [83]. Mutants with reduced activity of the IIS signaling component AGE-1, the *C. elegans* homolog of mammalian PI3K, also exhibit extended longevity [84, 85]. Remarkably, the longevity phenotype associated with IIS reduction is completely dependent on DAF-16, the FoxO homolog in *C. elegans*. Over the past 20 years, other longevity-regulating factors have been reported and for many of them, their functions on aging require direct or indirect involvement of DAF-16 activity. Overexpression of JNK-1 or CST-1 promotes lifespan extension by phosphorylating and activating DAF-16 [86, 87]. The loss of RLE-1, an E3 ubiquitin ligase that catalyzes DAF-16 polyubiquitination, extends lifespan [88]. In contrast, the deubiquitylase MATH-33 stabilizes DAF-16 and its loss of function inhibits the longevity phenotype of *daf-2* mutants [89]. In addition, transcription factors like HSF-1, SKN-1 and PQM-1 can function as cofactors for DAF-16 to synergistically mediate the extended lifespan induced by IIS inhibition [90–92]. Germline removal by laser microsurgery or genetic mutation of *glp-1* increases lifespan independent of IIS pathway but this benefit also relies on DAF-16 activity [93–96]. Although DAF-16 is not required for the lifespan extension effect of chronic calorie restriction in *eat-2* mutant [97], it is indispensable in longevity induced by calorie restriction in middle-aged worms or by intermittent fasting [98, 99]. At the molecular level, activation of DAF-16 by upstream signaling pathways leads to its translocation to the nucleus, where it binds to the chromosome and either activates or represses downstream genes in metabolism, autophagy, and stress response [100–102]. It is believed that the combined effects of DAF-16-mediated transcriptional changes leads to the lifespan extension in *C. elegans* [103].

#### Drosophila

Similar to worms, reduced IIS also promotes longevity in *Drosophila* and the extension of lifespan is dependent on the downstream transcription factor dFOXO, the fly homolog of FoxO and DAF-16 [104, 105]. However, unlike DAF-16, direct manipulation of dFOXO expression in certain tissues is sufficient to extend lifespan in *Drosophila*. Overexpression of dFOXO in adult *Drosophila* pericerebral fat body led to reduced neuronal expression of insulin-like peptides, as well as increased lifespan and fecundity [106, 107]. More recently, reduced IIS specifically in an astrocyte-like glia subtype was found to delay development and extend healthy lifespan in *Drosophila* in a dFOXO-dependent manner [108]. Although dFOXO is not required for the lifespan extension by dietary restriction [109], it mediates the negative effect of sugar-rich diet on longevity [110]. Mechanistically, studies have shown that increased fat storage and oxidative stress resistance are implicated in dFOXO-mediated lifespan extension [104, 106].

#### Mammals

Multiple long-lived mouse models have been created by genetic manipulations targeting growth hormone, IGF-1, IGF-1 receptor, insulin receptor, and insulin receptor substrate [111–116]. However, some of the modifications also lead to negative changes like growth defects and insulin resistance [112, 116]. FoxO3 was reported to be required for lifespan extension under dietary restriction condition [117], but its implication in IIS inhibition-induced longevity or the overexpression phenotype have yet to be elucidated.

Several genetic variations within *FOXO3* have been reported to be associated with human longevity in a number of geographical and ethnic groups [118–123]. These *FOXO3* single nucleotide polymorphisms (SNPs) in long-lived men and women were associated with lower prevalence of cancer and cardiovascular diseases along with higher insulin sensitivity [118, 124]. A study on two intronic *FOXO3* SNPs, *rs12206094*-T and *rs4946935*-A, revealed that both longevity-linked minor alleles are associated with enhanced promoter activity in cell-based assays and higher *FOXO3* mRNA expression in many human tissues [125]. Another intronic *FOXO3* SNP *rs2802292* G-allele was also found to be associated with increased *FOXO3* basal expression in population [126, 127]. It is proposed that these SNP-associated introns have a regulatory role on *FOXO3* expression [128]. More work is needed before a causal relationship between *FOXO3* and human longevity can be established.

### FoxO in brain development and CNS function

The mammalian brain is made up of two basic types of cells: neurons and glia. Glia can be further categorized into more specific types based on their morphology and functions, such as astrocytes, microglia, and oligodendrocytes. Neurons are the key players in the brain that serve as information processors and messengers while the star-shaped astrocytes support neuronal functions through facilitating synaptic signaling, supplying energy and nutrition as well as stress resolution. Microglia are the immune cells in the brain. They patrol the brain and respond to inflammatory stimuli. Oligodendrocytes, on the other hand, wrap around neuronal axons to form myelin sheaths, speeding up the transmission of electrical impulses along the exons.

FoxOs, as versatile transcription factors mediating various cellular processes, play important roles in the brain both during development and in adult. FoxO proteins show differential expression patterns in adult mouse brains. FoxO1 is strongly expressed in dentate gyrus and the ventral CA regions of the hippocampus as well as in striatum, whereas FoxO3 is more diffusely expressed throughout the brain including all hippocampal areas, cortex and cerebellum. FoxO6 shows significant expression across hippocampus, the amygdalohippocampal area and the shell of the nucleus accumbens. The expression of FoxO4, however, is very limited in the brain [129–131]. Genetic deletion of *Foxo* genes in the mouse brain results in varied phenotypes. Germline *Foxo3* null mice develop adult-onset auditory neuropathy making them partially deaf due to incorrectly positioned synapses within the cochlea [132]. *Foxo6* knockout mice are capable of learning but have a reduced ability to form long-term contextual and object recognition memories, probably due to lower dendritic spine density in hippocampal neurons [133]. Brain-specific deletion of *Foxo1* driven by the Nestin promoter results in increased anxiety in a forced swim test, contrary to the reduced anxiety in *Foxo3*-deficient mice [134]. Besides the neuronal phenotypes, ablation of *Foxo1* using the Synapsin 1 promoter driven Cre in mice leads to a blunted refeeding response, increased sensitivity to leptin and amino acid signaling, and increased locomotor activity [135]. Its deletion in sympathetic nervous system results in reduced catecholamine biosynthesis, lower energy expenditure, improved glucose clearance and increased bone mass [136].

FoxO proteins are well recognized for their ability to integrate upstream stress signals to initiate fate-determination programs such as survival or apoptosis, and this is also applicable to neurons. It was shown that in PC12 cells or primary cultured neurons, FoxO3 regulates apoptosis upon nerve growth factor (NGF) deprivation [137]. Further studies suggested that this response is downstream of FasL and JNK signaling pathways [138]. FoxO1 is also involved in neuronal apoptosis downstream of NMDA receptor signaling by controlling the expression of TXNIP [139]. Moreover, MST1 can phosphorylate both FoxO1 and FoxO3 in cerebellar granule neurons under ROS or NGF withdraw to stimulate their transcriptional activity leading to cell death [87, 140]. In contrast, methylation of FoxO3 by methyltransferase SET9 impairs its activity and contributes to granule cell survival [141]. In addition, FoxO3 was found to mediate neuronal autophagy, the suppression of which via either genetic knockdown or expression of an upstream microRNA displayed neuroprotective effects upon traumatic brain injury (TBI) [142, 143]. Interestingly, mitochondrial FoxO3 was also detected in hippocampal neurons where it was proposed to confer protection against glutamate toxicity during epilepsy [144].

Neural stem cells (NSCs) in the brain are capable of self-renewal or giving rise to new neurons and certain types of glial cells. Adult NSCs are mostly localized to the subventricular zone (SVZ) and subgranular zone (SGZ) where they are under tight control of proliferation and differentiation throughout life [145]. Knockout of *Foxo1/3/4* or *Foxo3* alone is sufficient to reduce the quiescence of NSCs, causing them to hyper-proliferate and, as a consequence, results in the depletion of the stem cell pool early in life. These FoxO-deficient NSCs also exhibit other phenotypes like decreased self-renewal and increased apoptosis and high ROS levels [146–148]. In addition, new-born neurons lacking FoxO exhibits altered dendritic morphology, increased spine density and aberrant spine positioning [149]. Mechanistically, Schäffner and colleagues found that proper NSC differentiation requires the involvement of autophagy machinery and this process is FoxO dependent [149].

FoxO’s function in the brain is not limited to neurons. FoxO3 was found to upregulate aquaporin 4 (AQP4), a water channel specifically expressed in astrocytes, to induce cerebral edema following TBI [150]. FoxO3 also keeps the astrocytes cell cycle in check and its expression significantly inhibits proinflammatory cytokine-induced astrocytes proliferation by activating cell cycle regulatory genes like p27^kip1^. Consistent with this observation, *Foxo3*-null mice exhibits severe astrogliosis [151]. In microglia, FoxO3 was shown to mitigate ROS by trans-activating antioxidant genes such as *Sod2* and *Cat*, and this process is under the regulation of deacetylase sirtuin-3 (SIRT3) [152]. Moreover, it has been reported that FoxO1-mediated p27^kip1^ expression is indispensable for oligodendrocyte regeneration after neonatal hypoxia [153], while it is also responsible for nitric oxide-induced apoptosis in the oligodendroglial culture [154].

Recent work in our lab further delineated the cell-type specific role of FoxO3 in the brain [155]. We showed that in primary cultures, the subcellular localization of FoxO3 in astrocytes is highly sensitive to the changes of upstream insulin signaling but the same treatments triggered no response of FoxO3 translocation in neurons. Using conditional knockout and AAV-mediated rescue of FoxO3 expression in mice, we confirmed the cortical astrogliosis phenotype reported in *Foxo3*-null mice [151] and demonstrated that the phenotype can be attributed to the cell-autonomous function of FoxO3 in astrocytes. Additionally, FoxO3 deficiency altered the expression of a subset of lipid-regulatory genes and the overall lipid profile in the cortex. Using primary astrocyte cultures, we found that *Foxo3* deletion not only impeded cellular ability to consume excess fatty acids, but also impaired mitochondrial function and blunted the capacity to uptake extracellular Aβ. All these dysfunctions could be rescued by astroglial AAV-FoxO3 overexpression. Given the critical role of astrocytes in maintaining brain homeostasis, loss of astrocytic FoxO3 may exacerbate neuronal vulnerability, particularly under pathological conditions such AD.

## Role of FoxO in age-associated diseases

### Type 2 diabetes mellitus

T2DM is a chronic metabolic disease that is most prevalent in older adults. In the past 20 years, the number of adults diagnosed with T2DM has more than doubled and it is currently the seventh leading cause of death in the United States. T2DM features hyperglycemia, insulin resistance and relative impairment in insulin secretion. The disease has both local and systematic manifestations and its pathophysiology closely involves FoxO. For example, the level and the subcellular localization of FoxO differ in pancreatic β cells at different stages of T2DM. Researchers observed predominantly inactive cytoplasmic FoxO1 in healthy β cells, but it is translocated to the nucleus in response to hyperglycemia. In advanced diabetes, FoxO1 disappears from β cells, accompanied with the loss of insulin signaling. It was proposed that FoxO1 preserves the balance of mitochondrial function by promoting the utilization of glucose over lipids. The dynamic behavior of FoxO1 reflects its role in mitigating β cell stress at an early stage but becomes exhausted when the disease proceeds [156–158]. Furthermore, FoxO is over-activated in many other organs including liver and skeletal muscles under insulin resistance conditions where it partakes clinical symptoms. Hepatic FoxO drives hyperglycemia by inducing the expression of G6Pase [159]. It can also promote hyperlipidemia by pushing the production and release of very low-density lipoprotein (VLDL)-triacylglycerol [34]. Moreover, FoxO’s regulation on bile acids through CYP8B1 is disrupted, resulting in more 12α-hydroxylated bile acids secretion. 12α-hydroxylated bile acids are less potent than non-12α-hydroxylated bile acids at inhibiting triacylglycerol and cholesterol synthesis, which contributes to the atherogenic lipid profile in T2DM [53]. Studies have shown that induction of diabetes in mice causes muscle atrophy due to upregulated ubiquitin–proteasome and autophagy clearance. However, triple knockout of *Foxo1/3/4* prevents the activation of protein degradation pathways and rescued the muscle loss [160].

### Alzheimer’s disease

AD is the most common form of dementia in the elderly population. Patients experience progressive loss of memory and other cognitive abilities, which isolates them from family and society and eventually leading to death. AD is ranked as the sixth leading cause of death in the United States and costs 18.6 billion hours of care from unpaid caregivers in 2019, a contribution valued at nearly 244 billion dollars [161].

**Fig. 4:**
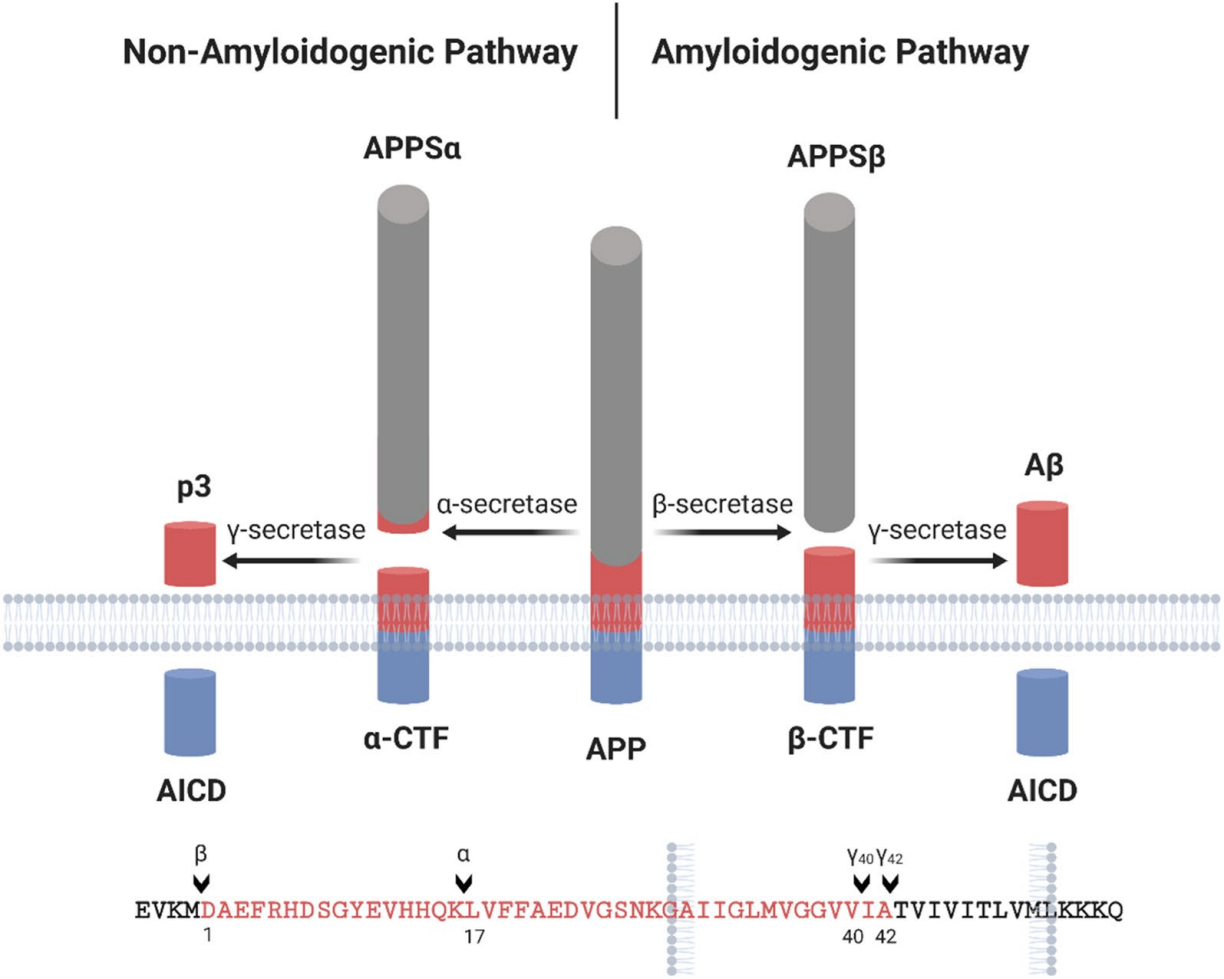
Proteolytic processing of APP. There are two distinct hydrolysis pathways in APP processing: amyloidogenic pathway (right) and non-amyloidogenic pathway (left). Sequential cleavage of APP by α-secretase and γ-secretase produces a soluble N-terminal peptide (sAPPα), a C-terminal APP intracellular domain (AICD) and a short fragment p3. This is the major secretory pathway with no Aβ production. On the other hand, cleavage by β-secretase and γ-secretase generates sAPPβ, AICD and Aβ [168, 169]. The cleavage sites of secretases are noted. The alternative cleavage of γ-secretase results in two Aβ species: Aβ_40_ and Aβ_42_

**Fig. 5:**
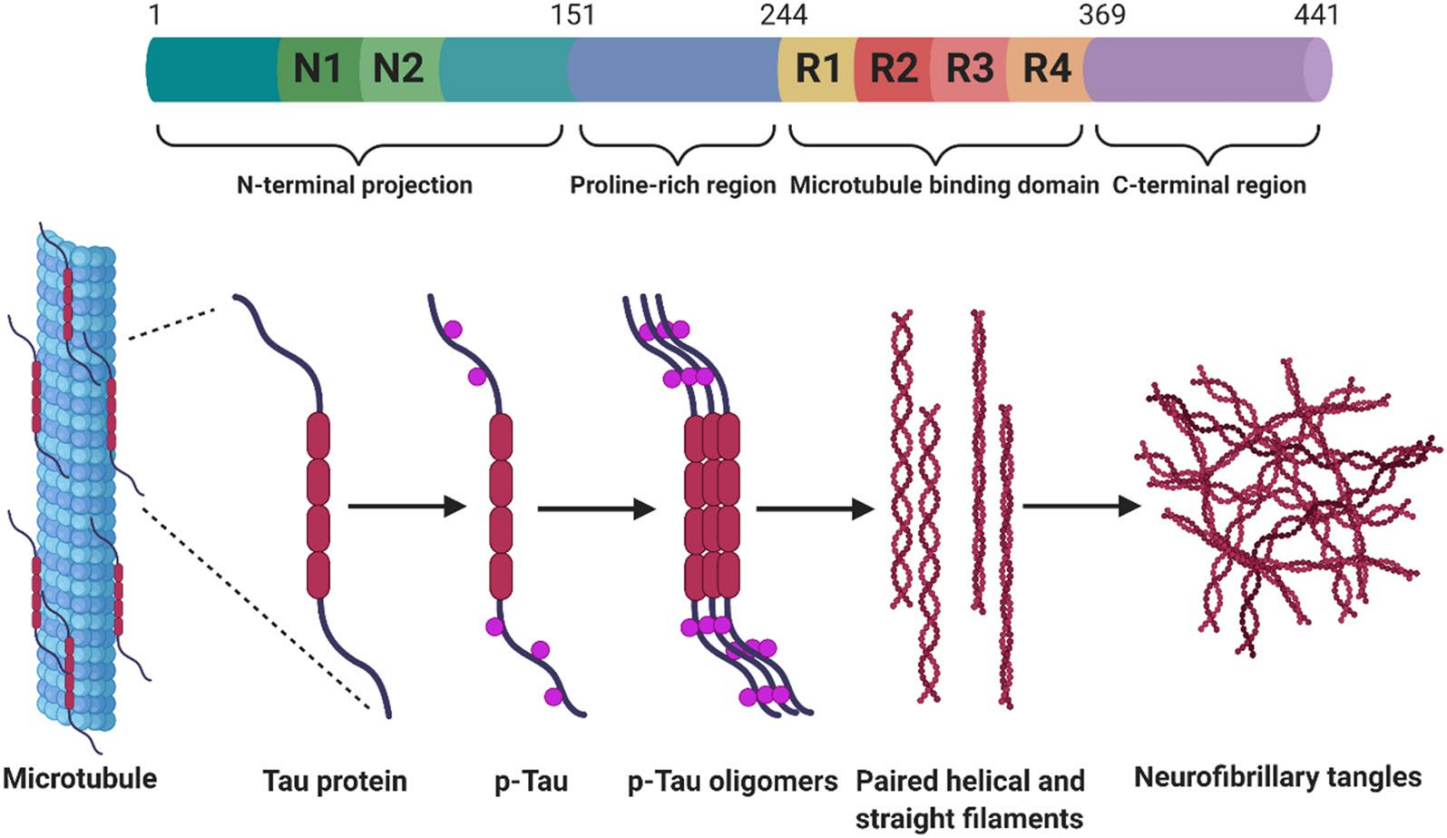
The functional domains of tau and its aggregation process. The alternative splicing of human *MAPT* gene generates six isoforms of tau proteins. They differ from each other by the presence or absence of one or two N-terminal inserts (N1, N2) and by bearing either three (R1, R3, R4) or four (R1, R2, R3 and R4) repeat domains. The structure of the longest isoform is shown here. The repeat domains are important for microtubule binding as well as aggregation under pathological conditions. It is believed that tau stabilizes microtubules in neurons and this process is regulated by kinases and phosphatases. Abnormally hyperphosphorylated tau (p-tau) dissociates from microtubules and form oligomers. The aggregation continues and leads to the assembly of paired helical filaments or straight filaments, and eventually forming neurofibrillary tangles [170, 171]

AD is pathologically characterized by the accumulation of extracellular senile plaques and intraneuronal neurofibrillary tangles (NFTs) in the brain. Senile plaques are aggregates of amyloid beta (Aβ) peptides while NFTs are mainly phosphorylated tau inclusions [162, 163]. Accordingly two major hypotheses have emerged based on the pathologic hallmarks [164, 165]. Aβ is derived from amyloid precursor protein (APP) through sequential cleavage. APP processing through the amyloidogenic pathway requires two enzymes, β-secretase and γ-secretase, which subsequently cleave APP leading to the release of Aβ peptides (Fig. 4). Aβ is rapidly degraded under normal conditions, however, in aged individuals or under pathological conditions, the clearance capacity is impaired, resulting in Aβ accumulation and aggregation. Aβ_40_ and Aβ_42_ are two common species of Aβ peptides, with Aβ_42_ being more prone to aggregation. Increased production of Aβ_42_ or a rise in ratio of Aβ_42_/Aβ_40_ from dysregulated cleavage often precede the formation of amyloid plaque. The amyloid hypothesis of AD pathogenesis proposed that the accumulated Aβ amyloid will develop into senile plaques, causing neurotoxicity and induction of tau pathology, eventually leading to neuronal cell death and neurodegeneration [166]. Alternatively, the tau hypothesis posits that the principal causative substance of AD is tau, a microtubule-associated protein that regulate the stability of tubulin assemblies. Hyperphosphorylated tau proteins under pathological conditions aggregate inside of neurons, disrupting the cellular homeostasis and causing neurodegeneration (Fig. 5). The amyloid hypothesis is strongly supported by the facts that amyloid deposits are a unique feature of AD and all identified genetic mutations in early-onset familial AD can be mapped to APP or presenilin 1/2, components of γ-secretase, and impact Aβ production. Nonetheless, the tau hypothesis agrees better with pathological staging of the disease and correlates with cognitive decline [166, 167]. The Aβ and tau pathologies trigger other neuropathological features that likely modulate disease progression. In particular, synapse dysfunction and loss show the strongest correlation with cognitive decline in AD patients. Neuroinflammation due to glial cell hyper-activation can impinge neuronal survival and function through secretion of toxic molecules and/or loss of trophic support.

Multiple studies implicate FoxO in AD pathophysiology although its exact role remains controversial. Several papers showed that FoxO proteins, especially FoxO3, mediate Aβ or AICD-induced apoptosis in primary cell cultures and in *Drosophila*, which leads to the loss of neurons or astrocytes [172–178]. FoxO3 has also been shown to be involved in Aβ-induced mitochondrial dysfunction in cultured neurons [179]. Qin and colleagues reported attenuation of amyloid pathology in a caloric restricted AD mouse model (Tg2576), and this is associated with activation of the insulin signaling pathway and downregulation of FoxO3 [180]. However, other reports demonstrated a protective role of FoxO in the brain. FoxO has been shown to protect neurons against Aβ-induced toxicity by upregulating stress resistance genes [181, 182]. Moreover, *Drosophila* FoxO (dFOXO) is essential for the maintenance of autophagy in the fly brain, and its reduction during aging promotes Aβ-induced neuronal dysfunction [183]. Interestingly, in mouse and monkey models of T2DM, researchers observed that reduced FoxO activities correlated with elevated Aβ and phosphorylated tau level, implicating a connection of T2DM and AD through FoxO [184]. Shi and colleagues proposed a two-sided role of FoxO3, with it being neuroprotective in the beginning but neurotoxic at later stages in AD [185]. They found that FoxO3, as a direct target of CDK5, promoted survival in HT22 cells under glutamate treatment by upregulating the oxidative stress resistant gene *Sod2* while it switched to a proapoptotic pathway after prolonged exposure in a BIM- and FASL-dependent manner. FoxO3 also mediated the glutamate induced Aβ production and consistently, the authors found activation and upregulation of FoxO3 in hippocampal neurons precede neurodegeneration and plaque formation in p25 transgenic mice [185].

Our study of FoxO3 in the 5xFAD mouse model supports a beneficial role of astrocytic FoxO3 against the amyloid pathology [155]. We found that Nestin-Cre mediated conditional knockout of *Foxo3* in 5xFAD mice significantly aggravated the plaque burden in the cortex compared to the littermate 5xFAD control. Increased plaque pathology was accompanied by enhanced synaptic loss and reduced recruitment of reactive astrocytes to the plaque location. Surprisingly, we also observed a blunted response of reactive microglia in the proximity of plaques. In contrast, AAV-mediated astrocyte-specific expression of FoxO3 in 5xFAD mice ameliorated the cortical plaque load, enhanced synapse number and also promoted the recruitment of both astrocytes and microglia to the plaques. Taken together, these results suggest that astrocytic FoxO3 modulates amyloid pathology through both a cell-autonomous effect and by influencing the recruitment of plaque-associated microglia.

### Other neurodegenerative diseases

In addition to AD, FoxO has been implicated in other neurodegenerative diseases like Parkinson’s disease (PD) and Huntington’s disease (HD). PD affects predominately dopaminergic neurons in the substantia nigra, and its symptoms include tremor, bradykinesia, limb rigidity and balance problems. During the course of PD, α-synuclein aggregates and accumulates in affected neurons to form Lewy bodies and ultimately causes neuronal death. Researchers have shown opposing roles of FoxO3 in dopaminergic neurons expressing α-synuclein. On the one hand, expression of a constitutively active form of FoxO3 induced degradation of α-synuclein and clearance of defective mitochondria. On the other hand, dominant negative forms of FoxO3 protected against death of dopaminergic neurons [186, 187]. Thus, while FoxO3 can protect cells from α-synuclein toxicity, it may also be responsible for neuronal death. In a rat model of PD, FoxO3 was found to inhibit the apoptosis in nigral dopaminergic neurons in a CDKN2D-dependent manner [188]. Likewise, the dFOXO function varies in different PD models in *Drosophila*. It ameliorates the mitochondrial defects and neuronal degeneration in *PINK1* null mutants while contributes to dopaminergic neurodegeneration in LRRK2-linked PD [189, 190].

HD is an autosomal dominant disease caused by an expanding poly-Q tract in the huntingtin (HTT) protein. It features abnormal involuntary movements, cognitive decline, and psychiatric disorders. Similar to the AD and PD studies, there are contrasting reports on FoxO’s role in HD. FoxO3 has been show in some studies to stimulate HD progression [191–193], whereas in other studies HD development was alleviated by FoxO [194, 195]. It is possible that the influence of FoxO3 activity differs with different stages of the disease. Interestingly, Vidal and colleagues unveiled a new mechanism of FoxO function in HD, where the loss of the unfolded protein response (UPR) transcription factor XBP1 leads to augmented expression of FoxO1, thus promoting autophagy and mutant HTT clearance [196].

Altogether, the published literature support a prominent role of FoxO in AD and other neurodegenerative diseases. However, its effect is likely dynamic and cell type-, context- and disease stage-dependent, which may explain the distinct and opposite results observed. Additional studies are needed to elucidate the underlying mechanisms and functional outcomes of FoxO in these diseases.

## Summary and perspectives

Aging is a complex process, and so are age-related diseases. FoxO transcription factors play a crucial role in both aging and age-related diseases, and thus could potentially serve as an underlying molecular link. Intriguingly, certain features of FoxO we learned from aging studies are consistent with those observed in disease context. For example, in *C. elegans* and *Drosophila*, the lifespan extension exerted by FoxO is likely the result of a combined autocrine and paracrine effects. DAF-2 is believed to primarily function in the nervous system. Mosaic worms with *daf-2* deficiency in a neuronal lineage and epidermis showed significantly increased lifespan [197]. In addition, expression of *daf-2* using neural promoters normalized the lifespan of *daf-2* mutants to control levels [198]. However, expression of *daf-16* in neurons only achieved 5–20% of lifespan extension in *daf-16; daf-2* animals. In contrast, restoration of DAF-16 activity in intestine, which is also the worm’s adipose tissue, is sufficient to extend their lifespan by 50–60% and it can also completely rescue the longevity defect of *daf-16* germline mutants [199]. Similarly, in *Drosophila*, limited activation of dFOXO in pericerebral fat body reduced the insulin-like peptide synthesis in neurons, and thus increases the lifespan of the animal [106]. It is possible that FoxO activity modulates lipid metabolism in these lipid storing cells, which in turn influence neuronal endocrine processes and lifespan in a non-cell autonomous manner. Strikingly, in mouse CNS, we found FoxO3 deficiency disrupts lipid metabolism in astrocytes, the glial cells which store and catabolize most lipids in the brain. In an amyloid mouse model, we showed that astrocytic FoxO3 not only function cell-autonomously, but also recruit microglia to engage amyloid plaques [155]. Although how FoxO3’s function in astrocytes affects cell–cell communication awaits further investigation, this interaction resembles what is observed in aging studies. Besides astrocyte-microglia interaction, it would also be interesting to understand whether astrocytic FoxO3 communicate with neurons and how these intercellular crosstalk modulate neuronal function and amyloid and tau/NFT pathology.

Accumulating evidence supports the existence of a shared risk for AD and T2DM [200]. The pathogenesis of AD is accompanied by significant disruption of metabolic pathways in the CNS. For example, AD brains suffer from oxidative stress and impaired glucose metabolism [201], and they take on features of insulin resistance [202, 203]. In addition, AD patients were found to carry more extensive islet amyloid, a pathogenic feature of T2DM, than non-AD controls [204]. Further, multiple reports implicate T2DM as a risk factor for AD development. Research in rodents demonstrated AD-like neuropathology in T2DM models and accelerated pathology development in AD models with T2DM-like metabolic changes induced by high-fat diet [205–209]. However, how peripheral insulin resistance modulates the central insulin signaling remains controversial. Studies in murine and primate models of T2DM showed an upregulated insulin signaling in affected brain, implying decreased FoxO activity under this condition [184]. We have shown that CNS FoxO3 deficiency leads to aggravated cortical amyloid pathology [155]. It has also been reported that FoxO mitigates β cell stress in early T2DM but such an effect is blunted when the disease proceeds [156, 157]. Age-associated decline of FoxO expression correlates with higher incidence of age-related diseases in the elderly population [155, 210]. Thus, proper levels of FoxO activity may be important not only for lifespan regulation but also confer protection against age-related diseases. Upregulation of FoxO activity thus holds therapeutic promise in the prevention or treatment of these age-related diseases. The fact that FoxO can be modulated by phosphorylation and other PTMs makes it an attractive target druggable by small chemicals. However, due to the complexity of the FoxO regulation and function, the level, site and duration of FoxO need to be tightly controlled. Therefore, in-depth understanding of each FoxO protein under different physiological and pathological conditions at molecular, cellular, and functional levels are needed to decipher their role in aging and disease to inform the development of future therapeutics.

## Data Availability

Not applicable.

## Abbreviations

T2DM: Type 2 diabetes mellitus
AD: Alzheimer’s disease
*C. elegans*: *Caenorhabditis* *elegans*
DBD: DNA-binding domain
DBE: DAF-16 family member-binding element
IRE: Insulin responsive element
PTMs: Post-translational modifications
NLS: Nuclear localization signal
NES: Nuclear export signal
TAD: Transactivation domain
IGFs: Insulin-like growth factors
IRS: Insulin receptor substrate
PI3K: Phosphatidylinositol 3-kinase
PIP3: Phosphatidylinositol (3,4,5)-trisphosphate
PDK1: Phosphoinositide-dependent kinase 1
PKB: Protein kinase B
ROS: Reactive oxygen species
JNK: C-Jun N-terminal kinase
AMPK: AMP-activated protein kinase
CDKs: Cyclin-dependent kinases
ATM: Ataxia telangiectasia mutated
ATR: ATM Rad3-related
HDACs: Histone deacetylases
SKP2: S-phase kinase associated protein 2
MDM2: Mouse double minute 2 homolog
PRMT1/6: Protein arginine methyltransferase 1/6
OGT: O-GlcNAc transferase
G6Pase: Glucose-6-phosphatase
PEPCK: Phosphoenolpyruvates carboxykinase
PDK4: Pyruvate dehydrogenase kinase 4
SREBP1c: Sterol regulatory element binding protein 1c
Scd1: Stearoyl-CoA desaturase I
LPL: Lipoprotein lipase
CPT1: Carnitine palmitoyltransferase-1
FAT: Fatty acid translocase
LDL: Low-density lipoprotein
PCSK9: Proprotein convertase subtilisin/kexin type 9
LDLR: LDL receptor
POMC: Proopiomelanocortin
AgRP: Agouti-related peptides
ARC: Arcuate nucleus
ATG: Autophagy-related
SOD2: Superoxide dismutase 2
IIS: Insulin/insulin-like growth factor-1 signaling
DBH: Dopamine-β-hydroxylase
NGF: Nerve growth factor
CGNs: Cerebellar granular neurons
TBI: Traumatic brain injury
NSCs: Neural stem cells
SVZ: Subventricular zone
SGZ: Subgranular zone
AQP4: Aquaporin 4
SIRT3: Deacetylase sirtuin-3
VLDL: Very low density lipoprotein
NFTs: Neurofibrillary tangles
Aβ: Amyloid beta
APP: Amyloid precursor protein
AICD: APP intracellular domain
PD: Parkinson’s disease
HD: Huntington’s disease
HTT: Huntingtin
UPR: Unfolded protein response
